# Uso da Inteligência Artificial Aplicada ao Eletrocardiograma para Diagnóstico de Disfunção Sistólica Ventricular Esquerda

**DOI:** 10.36660/abc.20240740

**Published:** 2025-04-29

**Authors:** Wilton Batista de Santana, Marcelo M. Pinto, Sandhi Maria Barreto, Murilo Foppa, Luana Giatti, Rohan Khera, Antonio Luiz Pinho Ribeiro

**Affiliations:** 1 Universidade Federal de Minas Gerais Belo Horizonte MG Brasil Universidade Federal de Minas Gerais (UFMG), Belo Horizonte, MG – Brasil; 2 Hospital de Clinicas de Porto Alegre Porto Alegre RS Brasil Hospital de Clinicas de Porto Alegre, Porto Alegre, RS – Brasil; 3 Yale University New Haven EUA Yale University, New Haven – EUA

**Keywords:** Inteligência Artificial, Insuficiência Cardíaca, Disfunção Ventricular Esquerda, Eletrocardiografia

## Abstract

**Fundamento:**

A insuficiência cardíaca é uma doença associada a importante morbimortalidade. O eletrocardiograma (ECG), um dos exames utilizados na avaliação da IC, é de baixo custo e amplamente disponível.

**Objetivo:**

Avaliar o desempenho de um algoritmo de inteligência artificial (IA) aplicado ao ECG na detecção de IC e o comparamos ao poder preditivo das alterações eletrocardiográficas maiores (AME).

**Métodos:**

Estudo transversal de acurácia diagnóstica. Todos os participantes são oriundos do Estudo Longitudinal da Saúde do Adulto (ELSA-Brasil), e possuíam ECG e ecocardiograma (ECO) válidos além de valores de probabilidade para disfunção sistólica do ventrículo esquerdo (DSVE) estimados pelo algoritmo. O desfecho avaliado foi fração de ejeção do ventrículo esquerdo (FEVE) < 40% ao ECO. Foram calculados sensibilidade, especificidade, valor preditivo positivo (VPP), valor preditivo negativo (VPN), razão de verossimilhança positivo (RVP), razão de verossimilhança negativa (RVN), *diagnostic odds ratio* (DOR) para o algoritmo e para as AME e área sob a curva ROC (ASC-ROC) para o algoritmo.

**Resultados:**

Na amostra final de 2567 indivíduos, a prevalência de FEVE < 40% foi de 1,13% (29 indivíduos). Os valores obtidos de sensibilidade, especificidade, VPP, VPN, RVP, RVN e DOR para o algoritmo foram de 0,690; 0,976; 0,244; 0,996; 27,6; 0,32 e 88,74, respectivamente. Para as AME, 0,172; 0,837; 0,012; 0,989; 1,09; 0,990 e 1,07, respectivamente. A ASC-ROC do algoritmo para predição de FEVE < 40% foi de 0,947 (IC 95% 0,913 – 0,981).

**Conclusão:**

A IA apresentou bom desempenho para detecção de DSVE e pode ser usada como ferramenta de triagem de DSVE.

## Graphical abstract

**Figura Central:**
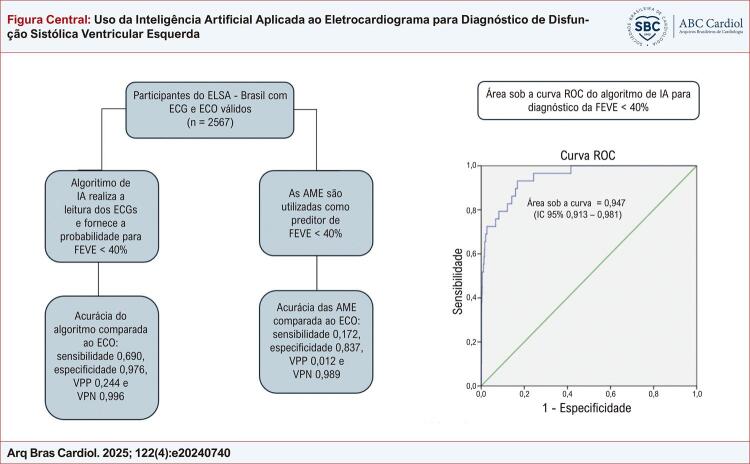
Uso da Inteligência Artificial Aplicada ao Eletrocardiograma para Diagnóstico de Disfunção Sistólica Ventricular Esquerda

Dados de acurácia de um algoritmo de IA e das AME para detecção da DSVE (FEVE < 40%). IA: inteligência artificial; AME: alterações maiores ao eletrocardiograma; DSVE: disfunção sistólica do ventrículo esquerdo; FEVE: fração de jeção do ventrículo esquerdo; ELSA: Estudo Longitudinal da Saúde do Adulto; ECG: eletrocardiograma; ROC: Receiver Operating Characteristic; VPP: valor preditivo positivo; VPN: valor preditivo negativo.

## Introdução

A insuficiência cardíaca (IC) está entre as 3 principais causas de doenças cardiovasculares no mundo.^1^ Trata-se de uma síndrome complexa com alta morbidade e custos para o sistema de saúde,^2-4^ tendo alta taxa de mortalidade intra-hospitalar.^1,5-7^ O ecocardiograma (ECO) é uma ferramenta de grande validade para o diagnóstico, permitindo o cálculo da fração de ejeção do ventrículo esquerdo (FEVE). Este parâmetro é fundamental para classificação em IC com fração de ejeção reduzida (ICFEr – FEVE < 40%), levemente reduzida ou intermediária (ICFEi – FEVE entre 40 e 49%) ou preservada (ICFEp – FEVE ≥ 50%) e tem implicações terapêuticas e prognósticas.^4,5^

Apesar de o ECO ser a principal ferramenta para diagnóstico e avaliação da IC, em países de média e baixa renda sua disponibilidade para uso disseminado à toda população elegível ainda é um desafio. Uma das estratégias para superar este problema é o aprimoramento de ferramentas mais acessíveis para avaliar pacientes em risco que se beneficiariam de propedêutica adicional.^6^ Dentre estas ferramentas, tradicionalmente o ECG, exame de baixo custo e amplamente disponível, é muito utilizado na avaliação inicial quando há suspeita de IC. Entretanto, para o diagnóstico dessa síndrome, tem acurácia limitada^3,4,7^ necessitando de aprimoramentos para prestar para este fim.

O uso e a disseminação da IA tem aumentado nos últimos anos, não sendo diferente na área da saúde.^8^ Dentre as áreas da IA, o aprendizado de máquina (AM) – ou *machine learning* – tem ganhado destaque nas aplicações na área médica.^9,10^ O número de estudos em IA aplicado à cardiologia aumentou significativamente nos últimos anos^11^ com aplicações possíveis na avaliação da idade cardiovascular,^12^ níveis séricos de potássio, detecção de fibrilação atrial (FA) silenciosa, detecção de cardiomiopatia hipertrófica,^13^ predição de hipotensão em pacientes de unidades de terapia intensiva (UTI)^14^ e diagnóstico de IC a partir da leitura do ECG.^15-24^

No presente trabalho foi avaliada a acurácia de um algoritmo de rede neural convolucional (RNC)^25,26^ utilizado para predizer, a partir da leitura do ECG, indivíduos que tenham DSVE, definida como FEVE < 40%. O desempenho do algoritmo foi comparado ao das AME, uma vez que, na prática clínica, tais alterações levam à suspeição de DSVE e ensejam extensão propedêutica, principalmente a realização de ECO.

Uma Rede Neural Convolucional (RNC ou *CNN –Convolutional Neural Network*, em inglês) nada mais é que um algoritmo de aprendizado profundo, ou *deep learning.* Esse tipo de rede capta o sinal de entrada, que pode ser uma imagem, e atribui pesos a vários dos seus aspectos. Dessa maneira, a RNC consegue diferenciar tais aspectos, o que é fundamental para a composição do sinal de saída. A arquitetura de tais redes é inspirada na do cérebro humano, mais especificamente do córtex visual. No caso da análise de ECG pode haver limitações como qualidade do traçado devido ao posicionamento de eletrodos e interferências, entre outros. Isso pode afetar a acurácia do modelo e sua utilização nas mais diversas condições de obtenção de um ECG.

## Métodos

### Desenho do estudo e participantes

Trata-se de um estudo transversal de acurácia diagnóstica. Os participantes são do Estudo Longitudinal do Adulto (ELSA-Brasil).^27^ Foram incluídos todos os indivíduos que tivessem ECG e ECO válidos, além dos dados de probabilidade de DSVE estimada pelo algoritmo de IA.

### Desenvolvimento da Rede Neural Convolucional

Para o desenvolvimento da rede, 385.601 ECGs foram pareados com seus respectivos ECOs. A validação interna foi realizada com internos do *Yale New Haven Hospital*. Já a validação externa foi realizada com indivíduos de cinco centros, entre eles o ELSA-Brasil. Foi utilizado um modelo de RNC baseado na arquitetura *EfficientNet-B3* para avaliação dos ECGs dos participantes. Esse tipo de arquitetura requer imagens de 300 x 300 pixels e inclui 384 camadas e tem mais de 10 milhões de parâmetros treináveis. Tal algoritmo foi desenvolvido e validado no *Yale New Haven Hospital* entre 2015 e 2021.

Tradicionalmente os algoritmos desenvolvidos para avaliação de ECG utilizam o sinal bruto, enquanto o algoritmo utilizado no nosso estudo utiliza a imagem do ECG. O algoritmo, após avaliação do ECG, informa um valor de probabilidade (0 -1) de haver ou não DSVE, sendo considerado um teste positivo quando a predição informada pelo algoritmo fosse maior do que 0,1 (10%). Como neste estudo o algoritmo foi utilizado para fins de triagem, se optou por este ponto de corte, que no artigo original ofereceu sensibilidade de 90%.^24^

### Obtenção do eletrocardiograma

Foi realizado o ECG convencional de 12 derivações utilizando um aparelho digital (Atria 6100, Burdick, Cardiac Science Corporation, EUA). As leituras de frequência cardíaca, duração, amplitude e eixos das ondas P, QRS e T, além dos intervalos QT, QTc e da dispersão de QT foram feitas de maneira automatizada. O Centro de Leitura (CL) de Eletrocardiografia localizado no Centro de investigação de MG (CI MG) foi o responsável pela leitura centralizada de todos os ECGs do ELSA, seguindo a padronização do Código de Minnesota.^27^ Para garantir a qualidade de uniformidade das análises a leitura e codificação dos exames, se criou um centro de leitura de ECG (CL-ECG), precedido pela visita a dois dos maiores centros de leitura de ECG o EPICARE na Carolina do Norte, EUA e o CARE, em Glasgow, na Escócia.^27^

### Obtenção do ecocardiograma

No ELSA-Brasil, o ECO foi realizado de maneira aleatória em 10% dos participantes, priorizando os participantes maiores de 55 anos. A aquisição de imagens foi feita por aparelhos Aplio XG (Toshiba), utilizando transdutor setorial de 2,5Hz. As imagens eram então encaminhadas digitalmente ao Centro de Leitura de Ecocardiografia de ELSA. Os exames foram obtidos por ecocardiografistas conforme protocolo de aquisição padronizado em consonância com recomendações vigentes para pesquisa. A leitura consistiu em análise qualitativa dos achados ecocardiográficos e mensuração de parâmetros quantitativos para definição dos desfechos de interesse do ELSA, incluindo: tamanho e geometria do ventrículo esquerdo (VE), tamanho do átrio esquerdo, função sistólica e diastólica do VE, presença de disfunção segmentar, lesões valvulares e degeneração fibrocálcica e espessura da gordura epicárdica.^27^ Considerou-se DSVE aqueles participantes que apresentaram FEVE < 40% ao ECO (pelo método Teichholz), exame de escolha para cálculo desse parâmetro. Dos métodos disponíveis para estimar a FEVE, o ECO é o mais acessível.

### Análise estatística

A descrição das variáveis foi feita utilizando mediana com intervalo interquartil para as variáveis contínuas de distribuição não-normal e frequência para variáveis categóricas. O teste utilizado para avaliar a normalidade dos dados foi o Kolmogorov-Smirnov e o nível de significância adotado foi p < 0,05.

Foram realizados cálculos das seguintes métricas: sensibilidade, especificidade, valor preditivo positivo (VPP), valor preditivo negativo (VPN), acurácia, *Diagnostic Odds Ratio* (DOR), razão de verossimilhança positiva (RVP), razão de verossimilhança negativa (RVN). Para o algoritmo também foi calculada a área sob a curva ROC (ASC-ROC) e foi utilizado o intervalo de confiança de 95%.

O software utilizado para as análises estatísticas foi o IBM SPSS *Statitiscs*, versão 21.

### Considerações éticas

O estudo original de onde parte nossa análise transversal aninhada foi aprovado pelo comitê de ética sob parecer no. ETIC 186/06.

Os termos de consentimento livre e esclarecido foram obtidos de todos os indivíduos em duas vias como prevê a resolução 196/96 do Conselho Nacional de Saúde e somente após a aposição de assinatura foram iniciados os procedimentos de avaliação.

## Resultados

**Figura 1:**
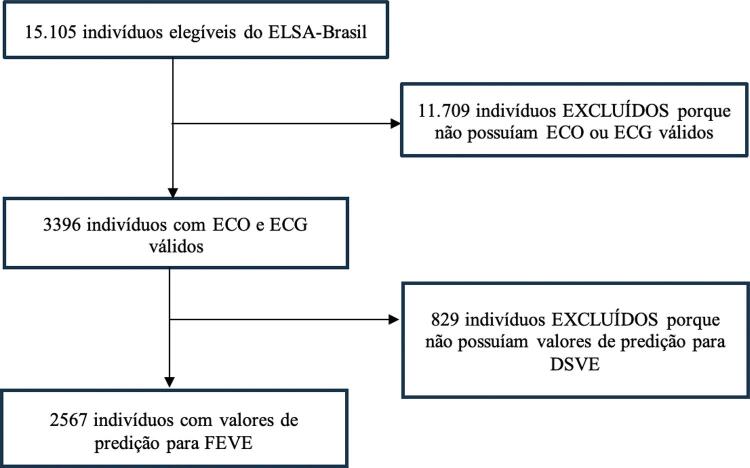
– Fluxograma de seleção dos participantes. ELSA: Estudo Longitudinal da Saúde do Adulto; ECO: ecocardiograma; ECG: eletrocardiograma; DSVE: disfunção sistólica do ventrículo esquerdo; FEVE: fração de ejeção do ventrículo esquerdo.

Após aplicados os critérios de seleção na amostra total do ELSA-Brasil, dos 15105 indivíduos, 3396 tinham ECO e ECG válidos. Desses, 2567 tinham ECO, ECG e informação de probabilidade de IC pelo algoritmo, havendo perda de 829 indivíduos. Provavelmente, essa perda ocorreu durante a transmissão para o centro onde os ECGs foram lidos pelo algoritmo. As características clínicas desses participantes se encontram na Tabela Suplementar 1. De forma geral, estes participantes apresentaram um perfil de risco cardiovascular mais grave quando comparado à população geral do ELSA-Brasil, mas semelhante ao perfil dos participantes incluídos no estudo. O fluxograma de seleção de pacientes está detalhado na Figura 1. As características clínicas dos participantes do estudo estão apresentadas na Tabela 1. A mediana da idade dos participantes foi de 62 anos tanto no grupo dos homens (45,4%) quanto nos das mulheres. Estas tiveram níveis séricos mais altos tanto de HDL-c quanto de colesterol total. Já entre os homens se verifica maior prevalência de dislipidemia, tabagismo, diabetes mellitus, acidente vascular cerebral, e doença cardiovascular autorreferida. A prevalência de FEVE < 40% foi de 1,13%. As características clínicas dos 15105 participantes do ELSA-Brasil estão disponibilizadas na Tabela Suplementar 2.

**Tabela 1:** – Características clínicas dos participantes do estudo

	População geral	Homens	Mulheres
Número	2567	1166 (45,4%)	1401 (54,6%)
Idade (anos), mediana e IQ (25-75)	52 (56 – 66)	62 (56,0 – 67,0)	62 (55,0 – 66,0)
Pressão arterial sistólica, mediana e IQ (25-75)	123,50 (113,00 – 136,50)	126,5 (116,37 – 139,50)	121 (110,00 – 134,50)
HDL colesterol (mg/dL), mediana e IQ (25-75)	52,90 (44,73 – 62,89)	46,54 (41,10 – 54,72)	58,35 (49,27 – 68,34)
Colesterol total (mg/dL), mediana e IQ (25-75)	198,10 (172,91 – 226,19)	192,29 (167,10 – 219,41)	202,94 (178,72 – 232,00)
Glicemia de jejum (mg/dL), mediana e IQ (25-75)	107,00 (100,00 – 117,00)	110 (103,00 – 121,00)	105 (98,00 – 114,00)
Dislipidemia (%)	55,7	50,9	59,7
Hipertensão arterial sistêmica (%)	49,5	53,6	46,1
Tabagismo (%)	10,0	11,8	8,4
Diabetes Melitus (%)	21,9	26,4	18,2
Doença Arterial Periférica (%)	5,6	5,3	5,9
Acidente Vascular Cerebral (%)	1,9	2,3	1,5
Doença Cardiovascular autorreferida (%)	10,9	14,1	8,3

A prevalência das AME está descrita na Tabela 2. As principais anormalidades foram alteração maior de ST-T isolada, alteração maior de onda Q (IAM antigo/prevalente) e o BRD completo, representando 6,5%, 3,9% e 3,1% das alterações, respectivamente.

**Tabela 2:** – Alterações eletrocardiográficas maiores e suas frequências, de acordo com o código de Minnesota

Alteração	Frequência (%)
Alterações maiores de ST-T isoladas	6,5
Alterações maiores de ondas Q (IAM antigo/prevalente)	3,9
Bloqueio de Ramo Direito completo	3,1
Prolongação maior do intervalo QT	2,2
Bloqueio de Ramo Esquerdo completo	1,0
Fibrilação Atrial/Flutter	0,9
Bloqueio intraventricular inespecífico	0,9
Hipertrofia Ventricular Esquerda mais alterações de ST-T	0,8
Alterações menores de onda Q mais alterações de ST-T (IAM prévio possível)	0,4
Pré-excitação ventricular	0,1
Marcapasso artificial	0,1
Bloqueio de Ramo Direito com bloqueio divisional anterossuperior	0,1
Padrão de Brugada	0,0
Bloqueio Atrioventricular de 3º grau	0,0
Bloqueio Atrioventricular de 2º grau	0,0
Fibrilação/assistolia ventricular	0,0
Taquicardia supraventricular	0,0

A distribuição da FEVE de acordo com a predição do algoritmo para DSVE está na Tabela 3. Dos 29 indivíduos com DSVE, o algoritmo identificou corretamente 20. Já na Tabela 4 está a distribuição da FEVE de acordo com a presença de AME. Dos 29 indivíduos com DSVE apenas 5 apresentavam AME.

**Tabela 3:** – Distribuição dos valores de predição do algoritmo de IA de acordo com a FEVE

		FEVE (%)	
		< 40 (1,13%)	≥ 40 (98,87%)
**Predição do algoritmo de IA (%)**	≥10	20	62
	<10	9	2476

IA: inteligência artificial; FEVE: fração de ejeção do ventrículo esquerdo.

**Tabela 4:** – Distribuição das alterações maiores ao ECG de acordo com a FEVE

		FEVE (%)	
		< 40 (1,13%)	≥ 40 (98,87%)
**Alterações maiores ao ECG**	**Presente**	5	413
	**Ausente**	24	2125

Fonte: Elaboração do autor.

**Figura 2:**
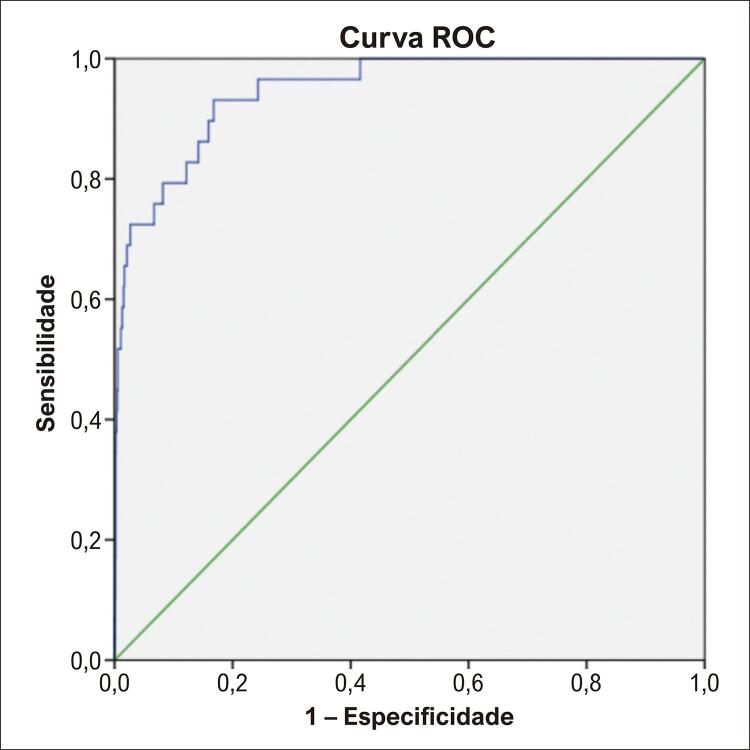
– Área sob a curva ROC do algoritmo de RNC para predição maior que 10% de FEVE < 40%. Fonte: Software SPSS, versão 21.

Os valores da sensibilidade, especificidade, VPP, VPN, RVP, RVN e DOR para o algoritmo e para as AME estão apresentados na Tabela 5. Para o algoritmo também foi calculada a ASC-ROC (Figura 2). O algoritmo apresentou valores maiores, quando comparado à AME, para sensibilidade (0,690 versus 0,172), especificidade (0,976 versus 0,837), RVP (27,6 versus 1,09) e DOR (88,74 versus 1,07). Para o algoritmo também foi calculado a ASC-ROC, 0,947 (0,913-0,981).

**Tabela 5:** – Sensibilidade, especificidade, VPP, VPN, RVP, RVN, DOR para o algoritmo de RNC e para as alterações maiores ao ECG. ASC-ROC para o algoritmo de RNC

Parâmetro	Algoritmo de RNC	Alterações maiores ao ECG
Sensibilidade (%)	69,0	0,172
Especificidade (%)	97,6	0,837
Valor preditivo positivo (%)	24,4	0,012
Valor preditivo negativo (%)	99,6	0,989
Razão de verossimilhança positiva	27,6	1,09
Razão de verossimilhança negativa	0,32	0,99
*Diagnostic odds ratio*	88,74	1,07
ASC-ROC	0,947 (IC 95% 0,913 – 0,981)	NA

NA: não se aplica; RNC: rede neural convolucional; ASC-ROC: área Sob a Curva – Receiver operating characteristic.

## Discussão

Neste estudo com 2567 indivíduos, o desempenho do algoritmo de IA para predição de DSVE se mostrou superior em relação às AME conforme demonstrado pelos testes de acurácia. Para a sensibilidade, o algoritmo obteve desempenho significativamente maior do que as AME, 69,0% *versus* 17,2%, respectivamente. Para especificidade, o algoritmo também apresentou melhor performance, 97,6% *versus* 83,7% para as AME. A RVP para o algoritmo foi de 27,6, aumentando expressivamente a probabilidade pós teste de DSVE na presença de teste positivo. Já para as AME o valor de RVP foi 1,09, ou seja, sua presença tem impacto quase nulo na probabilidade pós teste para DSVE. Outra métrica bastante expressiva para o algoritmo foi o DOR com valor de 88,74, significando que um indivíduo com DSVE tem 88 vezes mais chances de ser identificado pelo teste. Por fim, a ASC-ROC do algoritmo foi de 0,947, mostrando ter boa capacidade de discriminar indivíduos doentes dos não-doentes.

No nosso estudo, a prevalência de DSVE foi de 1,13% e o algoritmo de inteligência artificial (IA) desenvolvido apresentou uma sensibilidade de 69% e especificidade de 97,6%. A baixa prevalência resultou em um VPP pequeno, 24,4%, significando uma proporção muito grande de falsos positivos, e um VPN de 99,6%. Partindo de populações nas quais a prevalência de DSVE seja maior, por exemplo, em indivíduos sintomáticos ou com fatores de risco, o VPP será maior, resultando em uma maior poder de identificar indivíduos verdadeiramente doentes, ainda que às custas de um VPN menor, mas sem impacto significativo. Por exemplo, uma amostra hipotética na qual a prevalência de DSVE fosse 10% o VPP teria um aumento considerável de 24,4% para 79%, com mudança mínima no VPN (de 99,6% para 96%).

Supomos algumas razões para o algoritmo apresentar melhor desempenho quando comparado às AME. Primeiro e como já mencionado, as RNC são utilizadas no reconhecimento de padrões de imagens e avaliam alterações diferentes (ou padrões) das que tradicionalmente os médicos levam em consideração. É provável que a explicabilidade deste modelo não passe pela análise das alterações eletrocardiográficas tradicionalmente reconhecidas na prática clínica, dada a baixa acurácia dessas no nosso estudo. Além disso, o algoritmo consegue estabelecer relações entre esses padrões, conferindo maior poder às suas predições. Segundo, o algoritmo utilizado neste estudo é altamente específico, ou seja, ele foi projetado para avaliar o ECG (*input*) e fornecer um valor de predição (*output*). Isso aliado a *hardwares* mais robustos e à grande quantidade de dados disponíveis (*big data*) confere grande poder computacional culminando em análises mais precisas do ECG. Finalmente, uma RNC aprende a partir de milhares de ECGs, com mínima perda de dados. Por outro lado, um médico, ao longo do seu treinamento, é exposto a um número de ECGs muito menor e boa parte dos dados visualizados se perde devido a uma limitação natural da memória humana.

Outros estudos avaliaram o desempenho da IA para diagnóstico de DSVE (FEVE < 40%) apresentando também resultados semelhantes. Attia et al*.* em estudo realizado na Mayo Clinic que envolveu ECGs de mais 98 mil pacientes encontraram sensibilidade, especificidade e ASC-ROC de 86,3%, 85,7% e 0,93, respectivamente.^18^ Cho et al*.* avaliaram 3470 ECGs de 2908 pacientes encontrando sensibilidade, especificidade e ASC-ROC de 0,915; 0,911 e 0,961, respectivamente.^19^ Finalmente, Sangha et al*.*, utilizaram o algoritmo avaliado no nosso estudo aplicado a mais de 385 mil ECGs de 6 centros diferentes, um deles o ELSA-Brasil, obtendo sensibilidade, especificidade e ASC-ROC de 0,891; 0,900 e 0,949, respectivamente. Além disso, chegaram à conclusão de que as regiões de V2 e V3 foram as mais importantes para o cálculo de predição de DSVE.^24^

Nosso trabalho apresenta alguns pontos fortes. Primeiro, o ELSA-Brasil tem um banco de dados robusto, contando com 15105 indivíduos. Isso permitiu que tivéssemos um grande tamanho amostral (2567 participantes), conferindo solidez aos nossos achados. Segundo, as variáveis utilizadas têm fidedignidade uma vez que foram coletadas por equipe devidamente treinada nos CLs. Terceiro, no nosso estudo a prevalência de DSVE foi de 1,13%, portanto similar à prevalência no Brasil. Nos estudos avaliados para este trabalho, a prevalência de DSVE foi pelo menos 5 vezes maior do que na nossa população. O algoritmo mostrou bom desempenho, mesmo em um cenário de baixa prevalência da doença. Entretanto, a baixa prevalência pode superestimar o VPN obtido. Quarto, o ECG é um exame de baixo custo e amplamente disponível o que possibilitaria o uso do algoritmo em larga escala. No Brasil há cerca de 42 mil Unidades Básicas de Saúde (UBS) e mais de 460 Unidades de Pronto Atendimento (UPA). Em praticamente todas há um ou mais eletrocardiógrafos. De acordo com o Programa Nacional Telessaúde Brasil Redes existem 6 mil pontos de Telessaúde. Portanto, os ECGs das UBSs e das UPAs poderiam ser transmitidos aos pontos de Telessaúde e serem avaliados pelo algoritmo de IA, funcionando com um programa de triagem para IC. Aqueles indivíduos que fossem classificados como positivos pelo algoritmo, seriam então encaminhados para avaliação cardiológica e teriam prioridade para realização de ECO. Quinto, nosso estudo é um dos primeiros estudos avaliando o uso de IA para diagnóstico de IC em uma população brasileira. Sexto, ele compara a acurácia das AME à da IA no diagnóstico de DSVE. E, por fim, para desenvolvimento do algoritmo é necessária uma fase de treinamento na qual são pareados ECGs e ECOs para que o algoritmo detecte padrões e crie suas regras para o cálculo de probabilidade de DSVE. Portanto, o intervalo de tempo entre ECG e ECO deve garantir que esses exames reflitam a atual condição clínica do paciente. No nosso estudo o intervalo de tempo entre a realização do ECG e do ECO foi curto (semanas), garantindo que os exames avaliassem os indivíduos em condições clínicas muito semelhantes, senão iguais.

Nosso estudo apresenta algumas limitações. Primeiro, não sabemos como será o desempenho do algoritmo diante de ECGs que não sejam coletados com o mesmo rigor técnico do ELSA-Brasil. O correto posicionamento dos eletrodos cardíacos é fundamental para uma análise fidedigna. E, segundo, os exames analisados são de pacientes ambulatoriais, portanto desconhecemos o desempenho do algoritmo no contexto de emergência, sendo necessários estudos nesse sentido.

## Conclusão

A utilização de IA associada ao ECG tem potencial para impactar positivamente o cenário da IC no país. Sua utilização poderia permitir o diagnóstico precoce da IC bem como o tratamento, com potencial redução na mortalidade e morbidade (custos com internações, absenteísmo, aposentadorias por invalidez, melhora da qualidade de vida) por DCVs.

Como se trata de uma tecnologia nova, mais estudos são necessários para avaliar qual seria a acurácia desse algoritmo na análise de ECGs obtidos em situações de mundo real. Daí a necessidade de estudos prospectivos que validem a aplicação dessa tecnologia em diferentes cenários clínicos, garantindo sua aplicabilidade e impacto na prática médica diária.
