# Supplementary material for: Uso da Inteligência Artificial Aplicada ao Eletrocardiograma para Diagnóstico de Disfunção Sistólica Ventricular Esquerda
Source: Arq Bras Cardiol. 2025 Apr 29;122(4):e20240740. [Article in Portuguese] doi: 10.36660/abc.20240740 (PMC12108124; doi:10.36660/abc.20240740)
Supplement: Translation

# Translation: Use of Artificial Intelligence Applied to Electrocardiogram for Diagnosis of Left Ventricular Systolic Dysfunction

**DOI:** 10.36660/abc.20240740i

**Authors:** Wilton Batista de Santana, Marcelo M. Pinto, Sandhi Maria Barreto, Murilo Foppa, Luana Giatti, Rohan Khera, Antonio Luiz Pinho Ribeiro

**Affiliations:** 1 Brazil Universidade Federal de Minas Gerais (UFMG), Belo Horizonte, MG – Brazil; 2 Brazil Hospital de Clinicas de Porto Alegre, Porto Alegre, RS – Brazil; 3 USA Yale University, New Haven – USA

**Keywords:** Artificial Intelligence, Heart Failure, Left Ventricular Dysfunction, Electrocardiography

## Abstract

**Background:**

Heart failure (HF) is a disease associated with an important type of morbidity and mortality. The electrocardiogram (ECG), one of the tests used to evaluate HF, is low-cost and widely available.

**Objective:**

To evaluate the performance of an artificial intelligence (AI) algorithm applied to ECG to detect HF and compare it with the predictive power of major electrocardiographic alterations (MEA).

**Methods:**

This work is a diagnostic accuracy cross-sectional study. All participants were from the Longitudinal Study of Adult Health (Estudo Longitudinal da Saúde do Adulto - ELSA-Brasil) and presented a valid ECG and echocardiogram (ECHO). The algorithm estimated probability values for left ventricular systolic dysfunction (LVSD). The assessed endpoint was left ventricular ejection fraction (LVEF) <40% in the ECHO. Sensitivity, specificity, positive predictive value (PPV), negative predictive value (NPV), positive likelihood ratio (LR+), negative likelihood ratio (LR-), and diagnostic odds ratio (DOR) were determined for both the algorithm and the MEA. The area under the ROC curve (AUC-ROC) for the algorithm was calculated.

**Results:**

In the analytical sample of 2,567 individuals, the prevalence of LVEF <40% was 1.13% (29 individuals). The values obtained for sensitivity, specificity, PPV, NPV, LR+, LR-, and DOR for the algorithm were 0.690, 0.976, 0.244, 0.996, 27.6, 0.32, and 88.74, respectively. For the MEA, the values were 0.172, 0.837, 0.012, 0.989, 1.09, 0.990, and 1.07, respectively. The AUC-ROC of the algorithm to predict the LVEF <40% was 0.947 (95% CI: 0.913 – 0.981).

**Conclusion:**

The AI algorithm performed well in detecting LVSD and can be used as a screening tool for LVSD.

## Introduction

Heart failure (HF) is among the top three causes of cardiovascular disease (CVD) in the world.^1^ It is a complex syndrome with high morbidity and high costs for the health system,^2-4^ with a high in-hospital mortality rate.^1,5-7^ The echocardiogram (ECHO) is a highly valid tool for diagnosis, enabling the calculation of the left ventricular ejection fraction (LVEF). This parameter is essential for the classification of HF with reduced ejection fraction (LVEF <40%), slightly reduced or intermediate ejection fraction (HFrEF – LVEF between 40 and 49%), or preserved ejection fraction (HFpEF – LVEF ≥ 50%), and has therapeutic and prognostic implications.^4,5^

Although ECHO is the main tool for diagnosing and evaluating HF in low and middle-income countries, its availability for widespread use across the entire eligible population is still a challenge. One of the strategies to overcome this problem is the improvement of more accessible tools to evaluate at-risk patients who would benefit from additional propedeutics.^6^ Among these tools, the ECG, a low-cost and widely available test, is traditionally used in the initial assessment when HF is suspected. However, to diagnose this syndrome, the ECG has limited accuracy,^3,4,7^ requiring improvements to be used for this purpose.

The use and dissemination of AI has increased in recent years, and this is no different in the healthcare sector.^8^ Among the areas of AI, machine learning (ML) has stood out in applications in the medical field.^9,10^ The number of AI studies applied to cardiology has increased significantly in recent years^11^ with possible applications in the assessment of cardiovascular age,^12^ serum potassium levels, detection of silent atrial fibrillation (AF), detection of hypertrophic cardiomyopathy,^13^ prediction of hypotension in intensive care unit (ICU)^14^ patients, and diagnosis of HF based on ECG readings.^15-24^

The present work evaluated the accuracy of a convolutional neural network (CNN)^25,26^ algorithm, based on ECG readings, used to predict individuals who have left ventricular systolic dysfunction (LVSD), defined as LVEF <40%. The algorithm performance was compared to that of major electrocardiographic alterations (MEA) since, in clinical practice, such changes lead to the suspicion of LVSD and require extended investigations, including an ECHO exam.

A Convolutional Neural Network (CNN), also known as a Deep Learning algorithm, is essentially a type of deep learning network. This type of network captures the input signal, which could be an image, and assigns weights to various aspects of it. In doing so, the CNN is able to differentiate these aspects, which is crucial for forming the output signal. The architecture of such networks is inspired by the human brain, specifically the visual cortex. In the case of ECG analysis, there may be limitations, such as the quality of the tracing due to electrode positioning, interference, and other factors. These issues can impact the model’s accuracy and its application under diverse conditions for obtaining an ECG.

## Methods

### Study design and participants

This is a diagnostic accuracy cross-sectional study nested in the Longitudinal Study of Adult Health (*Estudo Longitudinal da Saúde do Adulto* – ELSA-Brasil).^27^ To be included in the study, individuals were required to have a valid ECG and ECHO in addition to LVSD probability data estimated by the algorithm.

### Development of the Convolutional Neural Network (CNN)

To develop the CNN, 385,601 ECGs were paired with their respective ECHOs. Internal validation was conducted with interns at the Yale New Haven Hospital. External validation was conducted with individuals from five centers, including ELSA-Brasil. A CNN model based on the *EfficientNet-B3* architecture was used to evaluate participants’ ECGs. This type of architecture requires 300 x 300 pixel images, includes 384 layers, and has more than 10 million trainable parameters. This algorithm was developed and validated at Yale New Haven Hospital between 2015 and 2021.

Traditionally, algorithms developed for ECG evaluation use the raw signal, while the algorithm used in our study uses the ECG image. After evaluating the ECG, the algorithm reports a probability value (0 -1) of whether or not LVSD is present. The test was considered positive when the prediction reported by the algorithm was greater than 0.1 (10%). As the algorithm was used for screening purposes in this study, this cutoff point was chosen, which offered a 90% sensitivity in the original article.^24^

### Obtaining the electrocardiogram

A conventional 12-lead ECG was performed using a digital device (Atria 6100, Burdick, Cardiac Science Corporation, USA). Readings of heart rate, duration, amplitude, and axes of P, QRS, and T waves, as well as QT, QTc intervals, and QT dispersion, were taken automatically. The Electrocardiography Reading Center (*Centro de Leitura* – CL), located at the MG Research Center (CI MG - UFMG), was responsible for the centralized reading of all ELSA-Brasil ECGs, following the Minnesota Code standardization.^27^ To guarantee the quality of uniform analysis, reading, and coding of exams, an ECG reading center (CL-ECG) was created, preceded by visits to two of the largest ECG reading centers, EPICARE in North Carolina, USA, and CARE, in Glasgow, Scotland.^27^

### Obtaining the echocardiogram

In the ELSA-Brasil study, ECHOs were randomly performed on 10% of the participants, with priority given to those over 55 years of age. The images were acquired using Aplio XG devices (Toshiba), using a 2.5Hz sector transducer. The images were then submitted to ELSA’s Picture Archiving and Communication System (PACS) and accessed by echocardiography CL (CI RS). Echocardiographers obtained the ECHOS according to a standardized acquisition protocol in line with current research recommendations. The reading consisted of the qualitative analysis of echocardiographic findings and measurements of quantitative parameters to define the ELSA endpoints of interest, including size and geometry of the left ventricle (LV), size of the left atrium, LV systolic and diastolic function, presence of segmental dysfunction, valvular lesions, and fibrocalcific degeneration, and epicardial fat thickness.^27^ Participants who had an LVEF <40% in the ECHO using the Teichholz method, the test of choice for calculating this parameter, were classified as having LVSD. Of the methods available to estimate LVEF, ECHO is the most accessible.

### Statistical analysis

Continuous variables with non-normal distribution were described using the median with interquartile range, while categorical variables were described by frequency. The test used to assess the normality of the data was the Kolmogorov-Smirnov test, and the significance level adopted was p < 0.05.

The following metrics were calculated: sensitivity, specificity, positive predictive value (PPV), negative predictive value (NPV), accuracy, diagnostic odds ratio (DOR), positive likelihood ratio (LR+), and negative likelihood ratio (LR-). The area under the ROC curve (AUC-ROC) was also calculated for the algorithm, and a 95% confidence interval was used.

The software used for statistical analyses was IBM SPSS Statistics, version 21.

### Ethical considerations

The original study from which the nested cross-sectional analysis is based was approved by the local ethics committee, logged under opinion no. ETIC 186/06.

The terms of free and informed consent were obtained from all individuals in two copies, as required by resolution 196/96 of the National Health Council, and procedures were only begun after the terms had been signed.

## Results

After applying the selection criteria to perform the ECHO, of the 15,105 ELSA-Brasil individuals, 3,396 presented a valid ECHO and ECG. Of these, 2,567 had ECHO, ECG, and HF probability information calculated by the algorithm; therefore, the sample suffered a loss of 829 individuals. This loss most likely occurred during transmission to the center where the algorithm read the ECGs. The clinical characteristics of these participants are shown in Supplementary Table 1. Overall, these excluded participants had a higher cardiovascular risk profile when compared to the ELSA-Brasil population, but a similar profile when compared to the participants included in the study. The patient selection flow diagram is detailed in Figure 1. Study participants’ clinical features are presented in Table 1. The median age of participants was 62 years of age in both the male (45.4%) and female groups. Women showed higher HDL-c and total cholesterol serum levels. Among men, there was a higher prevalence of dyslipidemia, smoking, diabetes mellitus, stroke, and self-reported CVD. The prevalence of LVEF <40% was 1.13%. The clinical features of the 15,105 ELSA-Brasil participants are available in Supplementary Table 1.

**Figure 1:** – Flow chart of the participant selection. ELSA-Brasil: Longitudinal Study of Adult Health; ECHO: echocardiogram; ECG: electrocardiogram; LVSD: left ventricular systolic dysfunction; LVEF: left ventricular ejection fraction.

The prevalence of MEA is described in Table 2. The main abnormalities were major isolated ST-T changes, major Q wave changes (old AMI), and full RBBB, representing 6.5%, 3.9%, and 3.1% of changes, respectively.

LVEF distribution according to the algorithm’s prediction for LVSD is shown in Table 3. Of the 29 individuals with LVSD, the algorithm correctly identified 20. LVEF distribution according to the presence of MEA is shown in Table 4. Of the 29 individuals with LVSD, only 5 had MEA.

The values obtained for sensitivity, specificity, PPV, NPV, LR+, LR-, and DOR for the algorithm and the MEA are provided in Table 5. The AUC-ROC was also calculated for the algorithm (Figure 2). The algorithm, when compared to MEA, presented higher values for sensitivity (0.690 versus 0.172), specificity (0.976 versus 0.837), PPV (27.6 versus 1.09), and DOR (88.74 versus 1.07). The AUC-ROC was also calculated for the algorithm 0.947 (0.913-0.981).

**Figure 2:** – Area under the ROC curve of the CNN algorithm for prediction greater than 10% of LVEF <40%. Source: Software SPSS, version 21.

## Discussion

In this study with 2,567 individuals, the performance of the AI algorithm to predict LVSD was superior to MEA, as demonstrated by the accuracy tests. For sensitivity, the algorithm performed significantly better than the MEA, 69.0% versus 17.2%, respectively. For specificity, the algorithm also showed a better performance, 97.6% versus 83.7% for MEA. The LR+ for the algorithm was 27.6, significantly increasing the post-test likelihood of LVSD in the presence of a positive test. For MEA, the LR+ value was 1.09; that is, its presence has almost no impact on the post-test likelihood for LVSD. Another very significant metric for the algorithm was the DOR, with a value of 88.74, meaning that an individual with LVSD is 88 times more likely to have a positive test, according to the algorithm. Finally, the AUC-ROC of the algorithm was 0.947, showing that it has a good ability to discriminate sick individuals from healthy individuals.

In our study, the prevalence of LVSD was 1.13%, and the artificial intelligence (AI) algorithm developed showed a sensitivity of 69% and a specificity of 97.6%. The low prevalence resulted in a low PPV of 24.4%, meaning a very high proportion of false positives and an NPV of 99.6%. To illustrate this consideration, in a hypothetical scenario of a population in which the prevalence of LVSD is higher, i.e., symptomatic individuals and those with risk factors, the PPV will be higher, resulting in an increased ability to identify truly diseased individuals, albeit at the expense of a slightly lower NPV, but without significant impact. For example, in a hypothetical sample where the prevalence of LVSD was 10%, the PPV would significantly increase from 24.4% to 79%, while the NPV would have a minimal decrease (99.6% to 96%).

We suggest some reasons why the algorithm has a better performance when compared to MEA. First, as already mentioned, the CNNs are used to recognize image patterns and evaluate different changes (or patterns) than those that doctors traditionally take into account. It is most likely that the explanatory nature of this model does not involve the analysis of electrocardiographic changes traditionally recognized in clinical practice, given their low accuracy in our study. Furthermore, the algorithm can establish relationships between these patterns, giving greater power to its predictions. Second, the algorithm used in this study is highly specific; that is, it was designed to evaluate the ECG (input) and provide a prediction value (output). This, combined with more robust hardware and the large amount of data available (big data), provides great computational power, culminating in more accurate ECG analyses. Finally, a CNN learns from thousands of ECGs with minimal data loss. Conversely, a doctor, throughout training, is exposed to a much smaller number of ECGs, and much of the viewed data is lost due to a natural limitation of human memory.

Other studies have evaluated the performance of AI in diagnosing LVSD (LVEF <40%) and have shown similar results. Attia et al., in a study carried out at the Mayo Clinic, which involved ECGs from over 98,000 patients, found sensitivity, specificity, and AUC-ROC of 86.3%, 85.7%, and 0.93, respectively.^18^ Cho et al. evaluated 3,470 ECGs from 2,908 patients, finding sensitivity, specificity, and AUC-ROC of 0.915, 0.911, and 0.961, respectively.^19^ Finally, Sangha et al., using the same algorithm evaluated in our study, applied to more than 385,000 ECGs from 6 different centers, one of which was ELSA-Brasil, obtained sensitivity, specificity, and AUC-ROC of 0.891, 0.900, and 0.949, respectively. Furthermore, they concluded that the V2 and V3 regions were the most important for calculating LVSD prediction.^24^

Our work presents some strengths. First, ELSA-Brasil has a robust database with 15,105 individuals. This allowed us to have a large sample size (2,567 participants), making our findings sound. Second, the variables used in our study are reliable, as they were collected by a team properly trained at the CLs. Third, in our study, the prevalence of LVSD was 1.13% and was thus similar to the prevalence in Brazil. In the studies evaluated for this work, the prevalence of LVSD was at least 5 times higher than in our population. Therefore, the algorithm showed good performance, even in a scenario of a low prevalence of the disease. However, the low prevalence may overestimate the NPV obtained. Fourth, the ECG is a low-cost and widely available test, which would allow the algorithm to be used on a large scale. In Brazil, there are around 42,000 Basic Health Units (BHUs) and more than 460 Emergency Care Units (*Unidade de Pronto Atendimento* – UPA). In practically all of these, there are one or more electrocardiographs. According to the National Telehealth Program, Brasil Redes, there are 6,000 telehealth points. Therefore, ECGs from BHUs and UPAs could be transmitted to telehealth points and evaluated by the AI algorithm, working with an HF screening program. Those individuals classified as positive by the algorithm would then be referred for cardiological evaluation and have priority for ECHO. However, our data presented here were validated in an outpatient scenario. Fifth, this is one of the first studies evaluating the use of AI to diagnose HF in a Brazilian population. Furthermore, sixth, it compares the accuracy of MEA to that of AI in diagnosing LVSD. Finally, for the development of the algorithm, a training phase is required in which ECGs and ECHO are paired so that the algorithm can detect patterns and create its rules for calculating the probability of LVSD. Therefore, the time interval between the ECG and ECHO must ensure that these tests reflect the patient’s current clinical condition. In our study, the time interval between the ECG and ECHO was short, ensuring that the tests assessed individuals in very similar if not identical, clinical conditions.

Our study has some limitations. First, we do not know how the algorithm will perform when faced with ECGs not collected with the same technical rigor as ELSA-Brasil. The correct positioning of cardiac leads is essential for a reliable analysis. Second, the exams analyzed in this study were from outpatients; therefore, we do not know how the algorithm will perform in emergency scenarios, and further studies in this regard are necessary.

## Conclusion

The use of AI associated with ECG has the potential to impact the HF scenario in Brazil positively. Its use could allow for an early diagnosis of HF as well as its treatment, with a potential reduction in mortality and morbidity (hospitalization costs, absenteeism, disability pensions, improvement in quality of life) due to CVDs.

Since this is a new technology, further studies are needed to assess the accuracy of this algorithm in analyzing ECGs obtained in real-world situations. Hence, there is a need for prospective studies to validate the application of this technology in different clinical scenarios, ensuring its applicability and impact on daily medical practice.

**Table 1:** – Study participants’ clinical features

	General population	Men	Women
Number	2567	1166 (45.4%)	1401 (54.6%)
Age (years), median and IQ (25-75)	52 (56 – 66)	62 (56.0 – 67.0)	62 (55.0 – 66.0)
Systolic blood pressure, median, and IQ (25-75)	123.50 (113.00 – 136.50)	126.5 (116.37 – 139.50)	121 (110.00 – 134.50)
HDL cholesterol (mg/dL), median and IQ (25-75)	52.90 (44.73 – 62.89)	46.54 (41.10 – 54.72)	58.35 (49.27 – 68.34)
Total cholesterol, mg/dL median and IQ (25-75)	198.10 (172.91 – 226.19)	192.29 (167.10 – 219.41)	202.94 (178.72 – 232.00)
Fasting blood glucose (mg/dL), median and IQ (25-75)	107.00 (100.00 – 117.00)	110 (103.00 – 121.00)	105 (98.00 – 114.00)
Dyslipidemia (%)	55.7	50.9	59.7
Systemic arterial hypertension (%)	49.5	53.6	46.1
Smoking (%)	10.0	11.8	8.4
Diabetes Melitus (%)	21.9	26.4	18.2
Peripheral artery disease (%)	5.6	5.3	5.9
Stroke (%)	1.9	2.3	1.5
Self-reported cardiovascular disease (%)	10.9	14.1	8.3

**Table 2:** – Major electrocardiographic alterations and their frequencies, according to the Minnesota code

Alteration	Frequency (%)
Isolated major ST-T alterations	6.5
Major Q wave alterations (old/prevalent AMI)	3.9
Complete right bundle branch block	3.1
Major QT interval prolongation	2.2
Complete left bundle branch block	1.0
Atrial fibrillation/Flutter	0.9
Nonspecific intraventricular block	0.9
Left ventricular hypertrophy plus ST-T alterations	0.8
Minor Q wave changes plus ST-T changes (possible previous AMI)	0.4
Ventricular pre-excitation	0.1
Artificial pacemaker	0.1
Right bundle branch block with anterior superior divisional block	0.1
Brugada pattern	0.0
3rd-degree atrioventricular block	0.0
2nd-degree atrioventricular block	0.0
Ventricular fibrillation/asystole	0.0
Supraventricular tachycardia	0.0

**Table 3:** – Distribution of AI algorithm prediction values according to LVEF

		LVEF (%)	
		**< 40**	**≥ 40**
**AI algorithm prediction LVEF (%)**	≥10	20	62
	<10	9	2476

AI: artificial intelligence; LVEF: left ventricular ejection fraction.

**Table 4:** – Distribution of major alterations on ECG according to the LVEF

		LVEF (%)	
		**< 40**	**≥ 40**
**Major ECG alterations**	**Present**	5	413
	**Absent**	24	2125

LVEF: left ventricular ejection fraction; ECG: electrocardiogram.

**Table 5:** – Sensitivity, specificity, PPV, NPV, LR+, LR-, and DOR for the CNN algorithm and major alterations in the ECG. AUC-ROC for the CNN algorithm

Parameter (%)	CNN algorithm	Major ECG alterations
Sensitivity (%)	69.0	0.172
Specificity (%)	97.6	0.837
Positive predictive value (%)	24.4	0.012
Negative predictive value (%)	99.6	0.989
Positive likelihood ratio	27.6	1.09
Negative likelihood ratio	0.32	0.99
Diagnostic odds ratio	88.74	1.07
AUC-ROC	0.947 (95% CI 0.913 – 0.981)	NA

NA: not applicable; CNN: Convolutional Neural Network; AUC-ROC: Area under the Receiver Operating Characteristic curve; ECG: electrocardiogram.
